# *Foundations for Literacy*: An Early Literacy Intervention for Deaf and Hard-of-Hearing Children

**DOI:** 10.1093/deafed/enu022

**Published:** 2014-08-14

**Authors:** Amy R. Lederberg, Elizabeth M. Miller, Susan R. Easterbrooks, Carol McDonald Connor

**Affiliations:** ^1^Georgia State University; ^2^Arizona State University

## Abstract

The present study evaluated the efficacy of a new preschool early literacy intervention created specifically for deaf and hard-of-hearing (DHH) children with functional hearing. Teachers implemented *Foundations for Literacy* with 25 DHH children in 2 schools (intervention group). One school used only spoken language, and the other used sign with and without spoken language. A “business as usual” comparison group included 33 DHH children who were matched on key characteristics with the intervention children but attended schools that did not implement *Foundations for Literacy*. Children’s hearing losses ranged from moderate to profound. Approximately half of the children had cochlear implants. All children had sufficient speech perception skills to identify referents of spoken words from closed sets of items. Teachers taught small groups of intervention children an hour a day, 4 days a week for the school year. From fall to spring, intervention children made significantly greater gains on tests of phonological awareness, letter–sound knowledge, and expressive vocabulary than did comparison children. In addition, intervention children showed significant increases in standard scores (based on hearing norms) on phonological awareness and vocabulary tests. This quasi-experimental study suggests that the intervention shows promise for improving early literacy skills of DHH children with functional hearing.

Reading is crucial to academic achievement and future life success. Unfortunately, many children, including those who are deaf and hard of hearing (DHH), struggle to learn to read (Qi & Mitchell, 2012). Over the last two decades, policy makers, educators, and researchers have focused extensively on understanding how to improve reading outcomes for all learners. Based on a meta-analysis of research with young hearing children, the National Early Literacy Panel (NELP, 2008) concluded that one important avenue for addressing reading outcomes is to ensure that all preschoolers have foundational early literacy skills prior to elementary school (Shanahan & Lonigan, 2010). Consistent with the Simple View of Reading (Gough & Tunmer, 1986), reading researchers provide evidence for the importance of two types of foundational skills: code-based skills necessary for decoding words (e.g., phonological awareness, alphabetic knowledge, and print concepts) and meaning-based skills necessary to understand the decoded words and thus ideas (e.g., vocabulary and language comprehension). The NELP found that preschool interventions can improve both code-based and meaning-based skills in hearing children at risk for reading failure, and such improvements result in better reading outcomes during elementary school (Shanahan & Lonigan, 2010).

Whereas we have made considerable gains in our understanding of early literacy interventions for hearing children, we know much less about facilitating the development of these skills for DHH children (Easterbrooks & Beal-Alvarez, 2013; Schirmer & McGough, 2005). This is despite the well-documented fact that the majority of DHH children enter kindergarten behind their hearing peers in both code-based and meaning-based literacy skills (Lederberg, Schick, & Spencer, 2013). Unrelated to cognitive impairment, hearing loss can interfere with access to language if parents are not fluent in sign language and typically leads to weaker language skills at all levels, which disrupt the process of learning to read at both the levels of decoding and language comprehension (Lederberg et al., 2013). Studies on reading interventions for DHH children have focused on school-age remedial interventions (see Easterbrooks & Beal-Alvarez, 2013; Trezek, Wang, & Paul, 2010 for reviews), yet preschool may be an especially important time for literacy development in DHH children. Lack of empirically validated early literacy interventions developed for DHH children and scant evidence regarding their effectiveness for DHH children is a challenge to professionals (Easterbrooks, Lederberg, & Connor, 2010).

This study was part of a research program focused on the development of an early literacy intervention for DHH prekindergarteners, called *Foundations for Literacy* (*Foundations*). We developed *Foundations* based on evidence about effective literacy interventions with hearing children but with specific adaptations to meet the needs of DHH children with functional hearing (i.e., those with sufficient speech perception to be able to understand at least some spoken words) because of our theoretical assumption that early literacy intervention should differ for DHH children with and without functional hearing (Lederberg et al., 2013). The aim of this study was to provide preliminary evidence of the intervention’s effectiveness with DHH children with functional hearing. Teachers implemented the yearlong *Foundations* intervention with 25 DHH children. We examined these DHH children’s gains in phonological awareness, alphabetic knowledge, and vocabulary from fall to spring of the school year compared to gains of DHH children matched on key characteristics, but who did not participate in the intervention (i.e., a business as usual control group). We also compared intervention children’s gains with gains made by hearing children in the tests’ normative samples.

## Challenges to DHH Readers

During the last two decades, many strides have been made that have influenced language and literacy outcomes for DHH children. These include benefits from Universal Newborn Hearing Screening (Lederberg et al., 2013), a greater chance to enter school with closer to age-appropriate language skills (Fitzpatrick, Crawford, Ni, & Durieux-Smith, 2011; Hayes, Geers, Treiman, & Moog, 2009), and changes in available audiological technology that has improved speech perception and auditory access to spoken language (Fitzpatrick et al., 2011). This access is primarily provided by cochlear implants (CIs) for children with severe to profound loss and digital hearing aids for those with less severe losses. These changes mean that the acquisition of spoken language is more feasible for many more DHH children than in the past. For example, two recent studies in different countries found that over 70% of children enrolled in preschool or early childhood programs for DHH children had functional hearing (Easterbrooks, Lederberg, Miller, Bergeron, & Connor, 2008; Hyde & Punch, 2011). DHH children with functional hearing may be acquiring spoken language alone or in combination with sign. In a 2008 national survey, Gallaudet Research Institute found that 53% of students with hearing loss used speech as their primary mode of communication, 35% used sign with speech, and 11% used sign alone.

We have proposed that two segments of the DHH population may learn to read through different processes (Easterbrooks & Beal-Alvarez, 2013; Lederberg et al., 2013). DHH children with functional hearing may learn to read by building on their spoken phonological abilities; children without functional hearing may learn to read through visually accessible processes that are not based on spoken phonology (e.g., see Haptonstall-Nykaza & Schick, 2007). This study focuses on the former group—DHH children with functional hearing.

DHH children with functional hearing face a number of challenges that interfere with learning to read (Ambrose, Fey, & Eisenberg, 2012; Lederberg et al., 2013; Nittrouer, Caldwell, Lowenstein, Tarr, & Holloman, 2012). Decreased access to spoken English results in incomplete phonological representations of phonemes and words. For example, “cats,” which is represented as /katz/ for a hearing child might be /ka/ for a DHH child because /t/ and /z/ are less salient, higher frequency, and thus more difficult to hear. As Perfetti (2007) suggests in the Lexical Quality Hypothesis, children who have small lexicons and incomplete or poor quality phonological representations of words are also less able to access or be aware of those representations. Because of language delays, DHH children may not know the words that they are learning to read. In addition, smaller lexicons may also result in weak phonological awareness. Additionally, some DHH children with functional hearing may be acquiring a sign language, which has a different phonological, grammatical, and lexical structure than English (Schick, Marschark, & Spencer, 2006).

DHH children with functional hearing appear to need the same foundational skills to learn to read as hearing children. Researchers have found that phonological awareness, alphabetic knowledge, and vocabulary correlate both concurrently and predictively with reading abilities in young children with CIs and hard-of-hearing children (Ambrose et al., 2012; Colin, Leybaert, Ecalle, & Magnan, 2013; Connor, Craig, Raudenbush, Heavner, & Zwolan, 2006; Easterbrooks et al., 2008; Nittrouer et al., 2012; Webb & Lederberg, 2014). These same studies also found the majority of DHH children showed deficits in these skills compared to hearing children, with wide individual differences. Therefore, there is a strong rationale for early intervention with DHH children with functional hearing that focuses on these skills.

## Early Literacy Instruction

Targeted interventions can improve foundational literacy skills in young hearing children and have long-term positive effects on preventing reading failure. Shanahan and Lonigan (2010) reported on a meta-analysis of 78 studies that showed that code-based interventions had moderate to large effects on improving phonological awareness and alphabetic knowledge of hearing preschoolers and kindergarteners. Code-based interventions that combined instruction on phonological awareness with instruction on alphabetic knowledge (including letter knowledge and early decoding strategies) had the largest effect size. A meta-analysis of 19 shared reading programs and 28 language enhancement interventions found large to moderate effect sizes for increasing hearing children’s oral language skills, particularly vocabulary (Shanahan & Lonigan, 2010).

Similarly, specialized early childhood preschools may accelerate growth in DHH children’s vocabulary growth (Hayes et al., 2009; Nittrouer, 2010). However, comparisons among early childhood classrooms have not identified any specific element or program that is associated with children’s language or literacy outcomes (Easterbrooks et al., 2010; Nittrouer, 2010). Whereas focus on language development is fundamental to early intervention of DHH children, instruction in other aspects of early literacy is not. Easterbrooks et al. (2010) found the amount of literacy instruction varied widely in early childhood classrooms for DHH children. In addition, teachers of DHH children have reported that they did not teach phonics or phonological awareness because they viewed spoken phonology as inaccessible or because they were not comfortable teaching it (Easterbrooks, Stephenson, & Mertens, 2006).

Unlike research with hearing children, there are almost no literacy intervention studies with DHH preschoolers—that is studies that use either experimental or quasi-experimental designs to examine the effectiveness of interventions focused on improving phonological awareness, alphabetic knowledge, and vocabulary. At the time of this writing, there is only one case study and four single-case design studies (*N* = 5–8) that suggest that phonics interventions may be effective in improving DHH preschoolers’ alphabetic knowledge or phonological awareness (Beal-Alvarez, Lederberg, & Easterbrooks, 2012; Bergeron, Lederberg, Easterbrooks, Miller, & Connor, 2009; Miller, Lederberg, & Easterbrooks, 2013; Smith & Wang, 2010; Tucci & Easterbrooks, 2014). Research with kindergarten and first-grade DHH children suggests that their phonological awareness skills (e.g., rhyme and phoneme segmentation skills) and alphabetic knowledge (e.g., understanding letter–sound associations, decoding words) improve when teachers use phonics programs developed for hearing children supplemented with visual support such as Cued Speech (Colin et al., 2013) or Visual Phonics (Trezek, Wang, Woods, Gampp, & Paul, 2007). Luckner and Cooke (2010) found no vocabulary intervention studies with DHH preschoolers. Fung, Chow, and McBride-Chang (2005) found that parents’ use of interactive storybook reading (i.e., dialogic reading) improved DHH school-age children’s receptive vocabulary. All but the Fung et al. study did not include control or comparison groups, so effect sizes were not calculated, and causal claims are limited by small sample sizes.

## Theoretical Foundation for *Foundations*

Theoretical and empirical research supported two assumptions that guided the development of *Foundations*. First, we assumed that the rich body of research on effective reading instruction for hearing children who are at risk for reading failures could form the initial basis for effective intervention for DHH children with functional hearing (Rayner, Foorman, Perfetti, Pesetsky, & Seidenberg, 2001; Schirmer & McGough, 2005; Shanahan & Lonigan, 2010). Empirically validated instructional strategies and comprehensive and balanced literacy prekindergarten programs for hearing children provided an initial framework for the development of *Foundations* (e.g., Lonigan, Purpura, Wilson, Walker, & Clancy-Menchetti, 2013; Phillips, Clancy-Menchetti, & Lonigan, 2008; Schwanenflugel et al., 2010). Theoretical and empirical research with hearing children also formed the basis for designing initial instructional strategies. For example, cognitive theories (e.g., Dual-Code theory; Sadoski & Paivio, 2001) and theories of early word reading (Ehri, 2014) suggest that targeted foundational skills would occur best in the context of instruction designed to build multimodal (visual, auditory, and kinesthetic) and semantic representations. Results from effective instruction for hearing children suggested that such representations are acquired through explicit instruction and multiple opportunities for practice embedded in developmentally appropriate meaningful, engaging activities (Schwanenflugel et al., 2010), including stories, language experiences, songs, and dialogic storybook reading (Lonigan et al., 2013).

Our second assumption was that we needed to adapt the intervention to the specific needs of DHH children with functional hearing. DHH children have incomplete phonological representations of phonemes and spoken words and weaker phonological processing skills, weaker language skills, and wider individual differences when compared to hearing children (Ambrose et al., 2012; Nittrouer et al., 2012). Thus, while adopting the literacy objectives of effective, integrated, code- and meaning-focused literacy prekindergarten programs for hearing children, *Foundations* is more systematic and its instruction is more explicit, multimodal, and intensive than might be used with hearing children. It provides visual and semantic support for the acquisition of phonemes crucial for children with weak speech and phonological processing skills associated with decreased access to the speech signal. The scope and sequence considers these children’s phonological representation of phonemes and spoken words and provides support for children who are language-delayed. Instruction uses multimodal strategies to build strong representations of letter(s)–sound correspondences and of the phonological structure, orthography, and meaning of words. It proceeds at a slower pace, incorporating carefully planned language and vocabulary requirements for activities. Additional instructional strategies addressing weak speech perception include using acoustic highlighting and emphasizing attention to lipreading cues (Easterbrooks & Estes, 2007). Visual representations (e.g., signs, fingerspelling, gestures, pictures, and Visual Phonics) are provided to support spoken English information to varying degrees depending on children’s needs. Teachers preteach vocabulary used in literacy and phonological awareness activities. To provide effective language stimulation, much of the instruction is embedded in language-rich activities. Finally, differentiation or individualization of instruction to the wide variation of language and phonological processing skills observed for children who are DHH is integral to the design.

## Present Study

The present study is part of a research program implemented over 5 years. A research team, which included teachers of the deaf and researchers, developed *Foundations* using an iterative design process. During the first year, we collected data on phonological awareness, alphabetic knowledge, and vocabulary in DHH children in one large metropolitan area and observed these children’s 11 self-contained classrooms prior to intervention (Easterbrooks et al., 2008, 2010). These studies indicated that teachers in these classrooms varied widely in their language and literacy practices and that DHH children ended the school year with inadequate gains in phonological awareness, alphabetic knowledge, and vocabulary.

During the spring of the first year, we conducted a short-term single-case design study to assess the effectiveness of a semantic association instructional strategy for teaching alphabetic knowledge (Bergeron et al., 2009), as well as piloting other components of the intervention. We then designed the overall structure of *Foundations* as a multicomponent, integrated, code-focused, and meaning-focused intervention to be implemented 1hr a day, 4 days a week over the school year.

The primary goal of the present study was to evaluate the efficacy of *Foundations* in facilitating early literacy skills in DHH children with functional hearing. We used a quasi-experimental design comparing the learning of children taught with *Foundations* with those who were not. During Years 2–4 of our research program, research teachers implemented *Foundations* with small groups of DHH children with functional hearing (*n* = 20). During Year 5, a research teacher and a classroom teacher team taught two small groups of children (*n* = 5). Each year, particular activities were enhanced based on the information gathered throughout the previous year. However, the overall structure, goals, and instructional strategies remained the same. We purposely chose two schools that represented different communication philosophies to ensure that *Foundations* could be implemented within different language learning environments. In one school, teachers and children used only spoken language. At the other school, teachers and children communicated with conceptually based English signs with and without spoken language, as well as American Sign Language (ASL). Thus, over the 4 years, teachers implemented *Foundations* with 25 DHH children with functional hearing (intervention children). During the 4 years of implementation, we continued to collect data in the fall and spring at seven other schools where *Foundations* was not implemented. At these schools, there were 33 children who met eligibility criteria for the intervention but who had teachers who did not use *Foundations* as their literacy instruction (comparison children).

Our primary goal was to evaluate the efficacy of *Foundations* in facilitating early literacy skills in DHH children with functional hearing. We addressed two research questions:

1. To what extent do children taught with *Foundations* show accelerated learning from fall to spring compared to hearing children? We hypothesized that intervention children would have these accelerated gains because researchers have found that effective early childhood programs result in such accelerated gains in DHH children (Hayes et al., 2009; Nittrouer, 2010).
2. Do children taught with *Foundations* (intervention children) demonstrate greater gains in phonological awareness, alphabetic knowledge, and vocabulary than do their peers who were not taught with *Foundations* (comparison children)?

## Method

### Participants

Participants had to meet the following eligibility criteria: (a) the ability to understand at least some spoken words solely through audition—defined as a score of 3 (some word identification) or 4 (consistent word identification) on the *Early Speech Perception* test (ESP; Moog & Geers, 1990); (b) chronological age between 3 years 8 months and 5 years 11 months as of September 1 of the school year; (c) no diagnosed or teacher-suspected additional severe disabilities such as autism or severe intellectual disability; (d) unaided hearing loss with a Better Ear-Pure Tone Average (BEPTA) of 50 dB or greater or at least one CI. Fifty decibels or greater was selected as a criterion because children with a moderate hearing loss have weaker speech perception and language abilities than those with less severe losses, even when children are appropriately fitted with hearing aids (Tomblin, Oleson, Ambrose, Walker, & Moeller, 2014).

#### Intervention children.

There were 25 children who were taught with *Foundations*. These children represented all children (with one exception) who met eligibility criteria at two schools during the years that the school administration agreed to participate in the study. We received such a commitment from a school that used solely spoken language for 4 years (*n* = 20 children taught). We received the same commitment from another school that used sign with and without spoken language for 2 years (*n* = 5).

#### Comparison children.

During Years 2–5, as part of our larger research program, we recruited DHH children from seven schools that had early childhood programs for children with hearing loss. These children were not taught with *Foundations*. Thirty-three of these children met the eligibility criteria for this study. All children who met eligibility criteria were included in the comparison group. No child contributed data for more than 1 year. Comparison children’s teachers used (a) spoken language only (*n* = 18 children), (b) simultaneous communication (SimCom: signed and spoken English; *n* = 11), or (c) a combination of SimCom and ASL (*n* = 4).

#### Demographics of intervention and comparison groups. 

Table 1 displays characteristics of both groups. Although intervention and comparison groups were not deliberately matched on these characteristics, they were similar along all but one dimension listed in Table 1. *t* tests and chi-square analyses indicated no significant differences for any of the dimensions listed in Table 1 except for proportion of children with CIs. Intervention children were more likely to have a CI (76%) than were the comparison children (46%), χ^2^(1) = 5.41, *p* < .05. This meant the comparison children were more likely to have a moderate to severe hearing loss and use hearing aids compared to the intervention children. As shown in Table 1, the groups were very similar in degree of hearing loss for those children without a CI measured by BEPTA (*M* = 65 dB).

**Table 1:** Demographic characteristics of intervention and comparison children

Characteristics	Intervention children	Comparison children
	Mean or %	Mean or %
Chronological age at pretest (months)	53.12 (5.71)	55.88 (6.16)
Age at identification (months)	11.84 (10.05)	12.34 (14.29)
Cochlear implants, % (*n*)	76% (19)	46% (15)
Age of implantation (months)	29.22 (11.84)	22.86 (7.77)
BEPTA for children with no CI	65.00 (13.9)	65.47 (10)
Gender, % girls	36% (9)	36% (12)
Deaf or hard-of-hearing parent	8% (2)	10% (3)
Ethnicity, % (*n*)		
White	56% (14)	56% (18)
African-American	32% (8)	25% (8)
Hispanic	4% (1)	16% (5)
Multiracial	4% (1)	4% (1)
Other	4% (1)	0
Maternal education level, % (*n*)		
Less than 12 years	0	6% (2)
High school graduate	16% (4)	25% (8)
Some college or technical	16% (4)	9% (3)
College graduate	40% (10)	41% (13)
Graduate school	24% (6)	13% (6)
Language used at home, % (*n*)		
English	84.0% (21)	66% (21)
Spanish	8% (2)	13% (4)
American Sign Language	16% (2)	8% (5)
Other language	0	6% (2)

*Note*. Intervention children (*n* = 25); comparison children (*n* = 33). Standard deviations are in parentheses for those variables with means. Number of children appear in parentheses for variables reported as proportions of sample. BEPTA = Better Ear-Pure Tone Average; CI = cochlear implant.

As a measure of verbal memory, children were also assessed with the Comprehensive Test of Phonological Processing—subtest 7 Memory for Digits (Wagner, Torgesen, & Rashotte, 1999). Scores were very similar in the two groups: intervention, *M* = 6.24, *SD* = 2.24; comparison, *M* = 6.57, *SD* = 2.88.

### Procedures

#### Assessment procedures and measures.

The children’s teachers supplied demographic information for each child, which was then checked by the children’s parents. Examiners administered a battery of language and literacy assessments in the fall and spring of each school year. All examiners were certified teachers of DHH children and had extensive experience in the language of the child’s school. Examiners administered each of the tests individually in a quiet, familiar room in school. The examiners were not informed of the goals of the study or the membership of the participants (i.e., intervention or comparison). Examiners used the communication mode of the school for the instructions for all tests and for all items on the reading and vocabulary tests. Examiners delivered the items on the speech perception and phonological awareness tests solely in spoken English with no accompanying sign or fingerspelling. Examiners followed required basal and ceiling rules for standardized tests. Test reliability statistics (as measured by Cronbach’s alpha) were calculated using data from our larger sample of 128 DHH children and are reported below. Examiners administered the following nine assessments:

#### Phonological awareness.

*Test of Preschool Emergent Literacy-Phonological Awareness* (TOPEL-PA, Lonigan, Wagner, & Torgesen, 2007) assesses 3- to 5-year-old children’s blending and elision of words, syllables, and phonemes. *The Phonological Awareness Test-2* (PAT, Robertson & Salter, 2007) contains four subtests that assess syllable segmentation, rhyme discrimination, initial phoneme isolation, and phoneme blending. Although the PAT was normed on 5- to 9-year-old hearing children, Webb, Schwanenflugel, and Kim (2004) found that the PAT can be used with hearing 4-year olds with appropriate modifications. We used the Webb et al. (2004) modifications, including two extra practice items, feedback, and a ceiling rule. Because of the off-level administration of the PAT, standard scores (based on hearing norms) were available only for the TOPEL-PA. Test reliability estimates were .96 for PAT and .89 for TOPEL. Item analysis suggests that both tests have good psychometric properties when used with DHH children with functional hearing (Webb & Lederberg, 2014).

#### Alphabetic knowledge.

On the researcher-created *Letter–Sound Identification Task* (Letter–Sound ID), children identify the sound(s) associated with the graphemes for 18 consonants, 3 digraphs, and 5 vowels (both long and short) for a total of 31 test items, including a 19th consonant as a trial item with feedback. *Woodcock-Johnson Tests of Achievement-III Letter-Word Identification* (WJ LWID; Woodcock, McGrew, & Mather, 2001) measures children’s letter-name knowledge and early word decoding. Examiners first ask children to name large type letters and then to read simple words. This means that for preschool children, the test primarily assesses letter-name knowledge. Test reliability estimates were .96 for the Letter–Sound ID and .84 for the WJ LWID. Standard scores were available for the WJ LWID.

#### Vocabulary.

*Woodcock-Johnson Tests of Achievement-III Picture Vocabulary* (WJ Vocab; Woodcock et al., 2001) and the *Expressive One Word Picture Vocabulary Test* (EOWPVT; Gardner, 2000) are expressive vocabulary tests. Children provide a signed or spoken word to label pictures. The Peabody Picture Vocabulary Test-III (PPVT; Dunn & Dunn, 1997) requires the child to select the correct picture out of four for a spoken (and signed when appropriate) word. Test reliability estimates were .82 for the WJ Vocabulary, .96 for EOWPVT, and .97 for PPVT. Standard scores were available for all three tests.

#### Descriptive measures.

The ESP (Moog & Geers, 1990) requires children to discriminate through audition alone among single words and/or multisyllable words with different stress patterns. Children must select correct referents of spoken words from closed sets of pictures/objects. The results are used to place children in four speech perception categories ranging from no pattern perception to consistent word identification. *Comprehensive Test of Phonological Processing—subtest 7 Memory for Digits* (Wagner et al., 1999) assesses children’s verbal memory by asking children to repeat strings of random numbers of increasing length. Examiners presented the items in the language of the children’s school.

### Intervention Procedures

Teachers instructed the children in small groups of one to three children (modal group size = 3), 4 days per week, 1hr per day, throughout the school year (September/October–May). Where more than three intervention children attended one school, school personnel assigned children to groups according to ability level. Teachers were certified teachers of the deaf and experts in the communication modality of the children’s school. During Years 2–4, one of two research teachers (i.e., teachers who were part of the research team) instructed 20 children. During the fifth year, a research teacher and a classroom teacher cotaught five children (divided into two small groups).

*Foundations* consists of 25-week-long instructional units with each unit containing 4-hr-long lessons. It is organized as an integrated curriculum where code-based and meaning-based learning objectives are frequently contained within the same instructional activity. *Foundations* begins with four introductory units in which teachers explicitly teach the instructional language needed to understand activities for the rest of the year. The other 21 instructional units have a common structure. Examples of instructional materials can be found in Supplementary Appendix A. Table 2 displays a summary of instructional activities.

**Table 2:** Percentage of time spent in major components of foundations (averaged across groups)

Component	Description	Average time, %	Target skills
Meaning-based activities^a^			
Miss Giggle letter–sound story	Telling and retelling the Miss Giggle stories	6.90	Vocab; L–S
Letter–sound language activity	Planning, doing, and recalling language activity that is related to the Miss Giggle stories	9.78	Vocab; L–S
Decodable word language activity	Language activity that relates to decodable word meaning	10.59	Vocab; PA
Dialogic storybook reading	Repeated readings of storybook with emphasis on vocabulary and child engagement	10.02	Vocab
Schedule	Teacher and child sequence the day’s activities on a sequencing chart	4.45	Vocab
Total spent in meaning-based activities		39.88	
Code-based activities^a^			
Reading activities	Child and or teacher decoded printed words (i.e., segmenting and blending letters or sound cards into words)	9.04	PA; Read
Phonological awareness activities	Explicit instruction in syllable segmentation, initial phoneme isolation, rhyming	10.88	PA
Practice activities	Individual and group practice on code-based skills, including PA, L–S fluency, reading	27.92	L–S, PA, Read
Total time spent in code-based activities		48.24	

*Note.* L–S = letter(s)–sound correspondences; PA = phonological awareness; Read = phonologically decoding print and sound cards; Vocab = vocabulary.

^a^Meaning-based activities are language-rich activities that include explicit focus on vocabulary. Code-based activities are those with explicit instruction in phonological awareness, letter–sound correspondences, and reading.

Each unit is organized around a story (referred to as the Miss Giggle Letter–Sound stories) that teachers use to explicitly teach letter(s)–sound correspondences and vocabulary in a language-rich narrative context (see Supplementary Appendix A). Each story focuses on one phoneme and the multiple ways to spell (i.e., encode) that phoneme. We include multiple spellings because we observed during the Year 1 single-case study that children sometimes produce two syllables for words such as *eat* (long e-, long a-t) when taught only single letter–sound correspondences (e.g., o-*o*). In contrast, when instruction included multiple spellings (o-*oa*; o-*ow*), children learned them readily and were able to correctly decode words such as *bow* and *boat*. Teachers present the story using illustrative sequence cards and pictures of targeted vocabulary. Related activities across the week include retelling the Miss Giggle story, planning, doing, and recalling a letter–sound language activity (e.g., the children making and flying paper airplanes).

We designed these activities to create a personally meaningful semantic association for the phoneme and to facilitate a multisensory representation of the letter(s)–sound correspondence. The activities also provided a fun context for children to engage in repeated practice in perceiving and producing individual phonemes. Each story is accompanied by a large sound card that displays the associated letters and a picture from the story activity. That picture acts as a mnemonic cue when children need to recall the phoneme (see Supplementary Appendix A). The picture is also used on small sound cards to represent the phoneme in subsequent reading activities (see Supplementary Appendix A). The advantage of the small concept cards is that there is a one-to-one correspondence between phoneme and cards (unlike letters and phonemes).

Each week, children receive explicit instruction on 6–10 enrichment vocabulary words selected from a list of words relevant to the weekly story. Instruction is accomplished through such evidence-based practices as explicit discussion of child-friendly word meanings accompanied by pictures, gestural representation (and sign when appropriate), and multiple opportunities to produce and comprehend the words in meaningful contexts—especially the weekly stories and subsequent language experiences (Schwanenflugel et al., 2010). Teachers differentiate instruction to children’s language level by using one of four levels of vocabulary (core, target, challenge, and extension—with pictures for the first three), as displayed in Supplementary Appendix A.

We follow instruction on letter–sound correspondences with emergent reading activities, using small sound cards or letters. After mastering the relevant letter(s)–sound correspondences, children engage in a decodable word language activity that provides repeated opportunities to hear, see, and produce a decodable word. For example, in one activity, after they have learned the letter–sound correspondences for *m* and *e*, the children play a question-and-answer game where the right answer is “me” (e.g., “Who has these eyes?” when shown a picture of the children’s eyes). These activities ensure children have strong semantic and phonological representations of the decodable word (Ehri, 2014). At the end of the decodable word language activity, teachers and students segment and blend the phonemes of the word (e.g., *me*) using the relevant small sound cards.

Following the introduction of a decodable word in language activities, children spent time reading those words in fun game-like reading activities. Objectives of these activities include phonological awareness and decoding. Reading decodable words provides repeated opportunities to segment and blend the phonemes of a word. Although reading is not a typical activity in prekindergarten programs, research suggests that children learn phoneme-level phonological awareness skills better when instruction includes letters (Shanahan & Lonigan, 2010), likely because letters serve as visual support for hard to discriminate phonemes.

Additional phonological awareness skills were developed through explicit instruction in syllable segmentation, initial phoneme identification, and rhyming. These activities frequently used the enrichment vocabulary from previous weeks to ease the language burden and focus attention on phonological awareness. Daily practice activities of previous skills included reviewing letter–sound correspondences using the large sound cards, letter(s)–sound fluency charts, reading connected text, and phonological awareness activities.

Teachers further reinforce literate language and vocabulary through daily storybook reading using dialogic reading, which is one of the best validated interventions for enhancing hearing and DHH children’s language skills (Fung et al., 2005; Shanahan & Lonigan, 2010). The goal is to increase the child’s active verbal interaction with an adult reading partner by asking questions, adding information, and prompting the child to increase the sophistication of descriptions of material in a picture book. Expansions of the child’s utterances and challenging questions from the adult encourage more sophisticated responses to create a true dialogue between teacher and child.

### Characteristics of Intervention Instruction

Teachers digitally video recorded their implementation of *Foundations*. To describe the intervention, we coded these recorded lessons in two ways.

#### Components of instruction.

First, graduate students coded the amount of time instructional groups spent in each of the major components of *Foundations* for all recorded lessons. In Table 2, we report the average percentage of the lessons spent in each of the major components of the intervention. Although to some extent most components included both meaning-based and code-based learning objectives, components can be classified into primarily one or the other. Observations revealed that *Foundations* was indeed a balanced intervention—almost equally divided between meaning-based and code-based activities (Table 2). To determine reliability, a second coder randomly selected one of the four lessons from a unit (approximately 25% of total lessons) to code. Average kappa across all years and groups was .79 with a range of .67–1.00, suggesting good interobserver reliability (Landis & Koch, 1977).

#### Procedural fidelity.

Graduate students rated the presence or absence of critical elements of instructional activities in the major components related to instruction on letter–sound correspondences, phonological awareness, and vocabulary (Table 3). Lessons were selected by randomly choosing 25% of the recorded lessons for each instructional group. To determine fidelity, we divided the number of lessons an essential element of an activity was present by the number of times that activity was observed. The overall high fidelity indicates that *Foundations* was implemented as intended (Table 3). Because of the explicit nature of the coding, we did not conduct interrater reliability on procedural fidelity.

**Table 3:** Procedural fidelity for teaching foundations for literacy (averaged across groups)

Attribute of lesson	Observed, %
Miss Giggle letter–sound story	
T reads/tells a story with phoneme, letter name, and large sound card	98
T writes model of target letter	93
T prompts S to imitate phoneme	95
S attempt to imitate T	98
Average for letter–sound story	96
Letter–sound story review	
Large sound card is visible to all S	92
T reviews story using sequence cards	89
T produces target phoneme	74
T prompts phoneme production	74
S attempt/produce phoneme	85
Average for story review	83
Letter–sound language activity	
S engage in sound concept activity	89
T models target sound during activity	98
S attempt/produce target sound	89
T provides articulatory feedback	89
Average for letter–sound activity	91
Language activity recall	
T and S recall language activity	100
T and S produce target sound	95
Average for language activity recall	98
Decodable word blending	
T uses small concept cards to make word	95
T identifies each phoneme while pointing to sound card/or letter for the word	86
T models blending word using continuous blending and sound cards	93
T prompts S to imitate	100
T or S points to cards while blending	98
Average for decodable word blending	94
Syllable segmentation	
T models segmenting word into sounds	95
T provides visual-kinesthetic representation of segments (pointing to visuals, tapping, clapping, etc.)	95
T prompts S to segment syllables	100
Syllable segmentation	
S attempts to segment syllables	100
T gives feedback	100
Average for syllable segmentation	98
Initial sound isolation	
T models initial sound by saying the word and its initial sound	80
T prompts S to give initial sound when presented with a word	100
S attempts to give initial sound	100
T gives articulatory feedback	100
Average for initial sound isolation	95
Rhyming	
T prompts S to listen or close their eyes and listen	80
T prompts S to say a rhyming word when presented with a target choices or to say yes or no when asked if a word pair rhymes	88
Average for rhyming	84
Practice books	
S attempt/produce target as S and/or T points to each letter	98
S move from page to page	98
Average for practice books	98
Letter–sound fluency chart	
Graphemes are visible on chart	100
S or T point to each grapheme	100
S attempts/produces grapheme	100
T immediately corrects and/praises	94
Average for fluency chart	99
Dialogic reading	
T asks at least three open-ended questions	86
T expands S’s language	90
T gives each S chance to respond	90
T targets vocabulary through questions, providing short definitions or picture cards	75
Average for dialogic reading	85

*Note.* S = student; T = teacher.

### Comparison “Business As Usual” Instruction

Comparison children were drawn from seven self-contained school programs (comparison classrooms). In the best experimental designs, researchers observe instruction in both comparison and intervention classrooms to identify how instruction differed. Because of economic constraints, we were not able to observe instruction in the comparison children’s classrooms. However, we did observe comparison and intervention classrooms prior to implementation of *Foundations* (i.e., in Year 1 of our research program) using the Early Language and Literacy Classroom Observation (ELLCO, Smith, Dickinson, Sangeorge, & Anastasopoulos, 2002). Our observations were reported in Easterbrooks et al. (2010). A high score on the ELLCO indicates optimal classroom instructional practices. Average total ELLCO score for the 13 teachers who were teaching in the school programs with comparison children was 67.5 (maximum possible is 107), which is considered below optimal. However, the classrooms varied widely with a range of 37–105 (*SD* = 19.45). Although these observations did not occur during the 4 years of data collection for the present study, they most likely reflect the instruction received by the comparison children because there was great stability in these programs—teachers for 75% of the comparison children were included in the ELLCO study, and our research team members who were well acquainted with these classrooms did not witness any major change in instruction across the 5 years. A comparison of the intervention and comparison classrooms revealed no significant differences in average ELLCO score prior to implementation to *Foundations* in Year 1, Mann–Whitney *U* test, *p* = .14.

We also informally surveyed the teachers in intervention classrooms in Year 1 (prior to implementation of *Foundations*) and comparison classrooms in Years 1–5 about their instructional practices related to early literacy. All teachers used a combination of teacher-created materials supplemented with curricula developed for hearing children. For example, one teacher who expressed the typical literacy approach said, “I did not use a specific curriculum. It was bits and pieces from here and there. I used Read It Once Again, TOTAL, Reading Milestones, Ready to Read, etc.” Other curricula used in classrooms included Children’s Early Intervention, and Animated Literacy.

## Results

Descriptive statistics for assessment scores of intervention and comparison children are displayed in Table 4.

**Table 4:** Mean standard and raw scores for intervention and comparison children on tests of phonological awareness, alphabetic knowledge, and vocabulary

Variable	Intervention group		Comparison group	
	Pretest	Posttest	Pretest	Posttest
Standard scores				
TOPEL-Phonological Awareness	85.12 (16.36)	93.00 (17.99)	84.57 (19.22)	85.48 (18.28)
WJ Letter-Word Identification	101.04 (12.68)	104.44 (16.14)	109.15 (13.53)	110.55 (14.79)
Expressive One Word Vocabulary	78.79 (13.02)	84.64 (15.70)	82.31 (13.49)	84.47 (14.63)
WJ Vocabulary	91.00 (14.64)	96.76 (10.69)	94.99 (16.29)	94.06 (10.90)
Peabody Picture Vocabulary	81.72 (14.43)	86.60 (16.40)	81.76 (13.86)	86.01 (14.30)
Raw scores				
TOPEL-Phonological Awareness	10.92 (4.86)	16.28 (6.11)	10.33 (7.68)	12.36 (7.64)
Phonological Awareness Test	3.92 (4.33)	19.36 (9.47)	7.52 (8.34)	14.88 (9.69)
Letter–sound Identification	4.80 (5.53)	16.32 (4.89)	9.39 (9.12)	14.85 (10.27)
WJ Letter-Word Identification	7.96 (4.61)	12.48 (6.29)	12.67 (6.75)	17.15 (8.68)
Expressive One Word Vocabulary	29.44 (10.24)	40.88 (11.85)	35.85 (11.70)	42.12 (11.85)
WJ Vocabulary	10.84 (3.8)	13.96 (2.79)	12.70 (3.97)	13.82 (2.98)
Peabody Picture Vocabulary	47.08 (16.41)	63.32 (21.12)	51.55 (17.51)	65.79 (20.06)

*Note.* TOPEL = Test of Preschool Emergent Literacy. Intervention children (*n* = 25); comparison children (*n* = 33). All tests had a mean standard score = 100; *SD* = 15 for the norming hearing sample. Standard deviations appear in parentheses.

We investigated the efficacy of *Foundations* in two ways. First, we analyzed fall to spring gains on standard scores for DHH children who received the intervention for those tests that were normed for hearing children. By definition, children making the gains typical of the hearing children in the norming sample would show no changes in standard scores from fall to spring. Therefore, if *Foundations* was efficacious, there should be a significant increase in standard scores. Repeated measures *t* tests revealed significant standard score gains by the intervention children for four of the five tests with standard scores (TOPEL-PA: *t*(23) = 2.913, *p* = .008; EOWPVT: *t*(23) = 4.506, *p* < .001; WJ Vocabulary: *t*(24) = 4.506, *p* < .001; PPVT: *t*(24) = 2.709, *p* = .012; WJ LWID: *t*(24) = 1.456, *p* = .158; see Table 3 for means and standard deviations). For example, on the TOPEL-PA, intervention children had an average gain of eight standard score points, an effect size (*d*) of .60. Effect sizes were even larger for expressive vocabulary (EOWPVT: *d* = .92; WJ Vocabulary: *d* = .74) and moderate for receptive vocabulary (PPVT: *d* = .31; Hill, Bloom, Black, & Lipsey, 2008). Although there was no acceleration of growth for WJ LWID, which for this age assesses letter naming, children did maintain typical gains (i.e., no decrease in standard score) and scored within the typical range for hearing children.

Second, as Lipsey et al. (2012) recommended, we estimated the intervention effect by “the difference between the covariate-adjusted means of the intervention and control samples” (p. 5). Specifically, we examined differential fall to spring gains for intervention and comparison children by conducting three multiple analyses of covariance (MANCOVAs)—one each for phonological awareness, alphabetic knowledge, and vocabulary. Spring raw scores of related tests were used as the dependent variables and fall raw scores of these tests were used as the covariate, with intervention status as the independent variable. Table 5 lists the covariate-adjusted means, mean difference, and effect sizes for the differences between intervention and comparison children for the seven dependent variables.

**Table 5:** Results of three multiple analyses of covariance examining gains by intervention and comparison children in phonological awareness, alphabetic knowledge, and vocabulary

Test	Group	Covariate- adjusted mean	Mean difference	Effect size (*d*)	Standard error	95% Confidence interval	
						Lower bound	Upper bound
Phonological awareness^a^							
TOPEL-Phonological Awareness	Intervention	16.464^a^	4.24	.302	0.867	14.726	18.202
	Comparison	12.224^a^			0.748	10.726	13.723
Phonological Awareness Test	Intervention	20.655^a^	6.76	.402	1.392	17.864	23.445
	Comparison	13.898^a^			1.200	11.492	16.304
Alphabetic knowledge^b^							
Letter–sound identification	Intervention	18.540^b^	5.28	.350	1.292	15.950	21.130
	Comparison	13.167^b^			1.113	10.935	15.398
WJ Letter-Word Identification	Intervention	15.410^b^	ns	ns	0.888	13.630	17.189
	Comparison	14.932^b^			0.765	13.399	16.465
Vocabulary^c^							
Expressive One Word Vocabulary	Intervention	43.311^c^	3.03	.257	1.201	40.902	45.720
	Comparison	40.279^c^			1.039	38.196	42.363
WJ Vocabulary	Intervention	14.527^c^	1.14	.396	0.254	14.017	15.037
	Comparison	13.389^c^			0.220	12.948	13.830
Peabody Picture Vocabulary	Intervention	66.278^c^	ns	ns	2.445	61.374	71.183
	Comparison	63.547^c^			2.115	59.305	67.789

*Note.* EOWPVT = Expressive One Word Picture Vocabulary Test; ns = nonsignificant; PAT = Phonological Awareness Test-2; PPVT = Peabody Picture Vocabulary Test-III; TOPEL-PA = Test of Preschool Emergent Literacy*-*Phonological Awareness; WJ LWID = Woodcock-Johnson Tests of Achievement-III Letter-Word Identification.

^a^Covariates appearing in the phonological awareness model are evaluated at the following values: TOPEL-PA pretest = 10.59, PAT pretest total = 5.9655.

^b^Covariates appearing in the alphabetic knowledge model are evaluated at the following values: WJ LWID pretest = 10.64, Letter–Sound ID pretest = 7.41.

^c^Covariates appearing in the vocabulary model are evaluated at the following values: EOWPVT pretest = 33.0862, WJ Vocab pretest = 11.90, PPVT pretest = 49.62.

The first MANCOVA showed that intervention children made significantly greater gains than did comparison children on phonological awareness skills, *F*(2,53) = 8.08, *p* < .001, Wilks’ lambda = .766. Follow-up analyses of covariance (ANCOVAs) showed that intervention children made more gains on both the PAT, *F*(1,52) = 17.95, *p* < .001, and the TOPEL-PA, *F*(1,52) = 41.63, *p* < .001, than comparison children. The second MANCOVA examined differences in alphabetic skills and revealed a significant advantage for children in the *Foundations* intervention, *F*(2,53) = 4.61, *p* < .05, Wilks’ lambda = .852. A post hoc ANCOVA revealed intervention children learned more letter–sound correspondences than did the comparison children, *F*(1,54) = 9.25, *p* < .004. The third MANCOVA showed that intervention children made significantly greater gains on vocabulary tests compared to comparison children, *F*(3,51) = 3.61, *p* < .02, Wilks’ lambda = .852. Follow-up ANCOVAs showed that intervention children made greater gains on the two expressive vocabulary tests—WJ-Vocabulary: *F*(1,54) = 10.99, *p* = .01 and EOWPVT: *F*(1,53) = 3.49, *p* = .067, but not on the PPVT: *F*(1,54) = 0.684, *p* = .41. As shown in Table 5, effect sizes of the intervention were similar across the three areas with an average of .35, indicating a moderate effect.

Because intervention and comparison groups differed in the proportion of children who had CIs, we conducted three one-way (audiological device) MANCOVAs that compared gains made by children with CI with those made by children who used hearing aids. There was no effect of audiological device on gains in phonological awareness: *F*(2,53) = 0.267, *p* = .75; alphabetic knowledge: *F*(2,53) = 0.405, *p* = .67; or vocabulary: *F*(3,51) = 0.06, *p* = .98. This is consistent with studies that found that children with CIs performed similarly to children with less severe hearing loss who used hearing aids (Fitzpatrick et al., 2011; Leigh, Dettman, Dowell, & Sarant, 2011).

## Discussion

Two broad foundational early literacy skills contribute to future reading success for both hearing and DHH children with functional hearing: code-based skills (phonological awareness and alphabetic knowledge) and meaning-based skills (vocabulary). Some struggling readers have difficulty with code-based skills, whereas others have difficulty with meaning-based skills (Snowling & Hulme, 2012). DHH children typically have difficulty with both (Lederberg et al., 2013). Although there is well-documented evidence that preschool interventions have been successful in ameliorating reading difficulties for struggling hearing readers (Shanahan & Lonigan, 2010), we have insufficient information to determine whether such interventions can support the development of DHH preschoolers. Overall, our results revealed that *Foundations* appeared to improve both code-based and meaning-based skills in children who are DHH with functional hearing.

### Efficacy

We used two indicators of efficacy—gains in standard scores and significantly greater gains in raw scores compared to comparison children who did not participate in *Foundations*. Both methods indicated that targeted experiences can facilitate development of phonological awareness, alphabetic knowledge, and vocabulary for DHH children with functional hearing. Standard scores moved from below average to close to the average of the hearing norming sample. Effect sizes were moderate and educationally important (Hill et al., 2008). This is the first quasi-experimental study that has indicated that school-based preschool interventions can improve these early literacy skills in DHH children.

#### Phonological awareness.

In the fall, the children were performing about 1 *SD* below hearing norms on a standardized measure of phonological awareness. This is similar to results in three recent studies with other samples of DHH children with CIs or who are hard of hearing (Ambrose et al., 2012; Dillon, de Jong, & Pisoni, 2012; Nittrouer et al., 2012). In the present study, comparison children ended the school year with the same below average standard score with which they started the school year, whereas intervention children showed significant gains. Taken together, these findings suggest that the majority of the current cohort of DHH children enter kindergarten behind their hearing peers on their awareness of the phonological structure of spoken words and that they do not show improvements without targeted intervention. This is not surprising given that DHH children have decreased auditory access that typically results in weaker phonological representations and smaller spoken word lexicons. However, the present results indicate that these barriers do not prevent them from being able to develop phonological awareness during the preschool years, provided they receive effective intervention. Gains on the PAT, which measured syllable segmentation, rhyming, and initial phoneme isolation, were consistent with those of a single-case design study that showed a functional relationship between instructional strategies included in *Foundations* and acquisition of these phonological awareness skills (Miller et al., 2013). Gains on the TOPEL-PA, which measures blending and elision skills, showed that instruction also improves these important phonological awareness skills in DHH children. Our results indicate that targeted and appropriate interventions can bring these skills nearer to age-typical for hearing children.

#### Alphabetic knowledge.

Intervention children increased their knowledge of letter–sound correspondences more than comparison children. Children taught using *Foun dations* knew, on average, 16 letter–sound correspondences by the end of the school year. This means that most children learned almost all of the 18 letter–sound correspondences included in the *Foundations* curriculum. This is also what evidence suggests is the optimal benchmark for the number of letters children should know by the end of preschool (Piasta, Petscher, & Justice, 2012). Consistent with previous research (Beal-Alvarez et al., 2012; Bergeron et al., 2009; Tucci & Easterbrooks, 2014), these results suggest that instruction that uses a personally meaningful semantic association strategy combined with repeated opportunities to practice can be effective in teaching letter–sound correspondences to DHH children. In contrast, *Foundations* did not result in accelerated learning of letter-names, as measured by either standard scores or compared to learning by comparison children. This may be because intervention and comparison children showed average to above-average skills on this assessment, in comparison to hearing norms. This is consistent with other studies (Ambrose et al., 2012; Easterbrooks et al., 2008) that also found age-appropriate skills in assessments of DHH preschoolers or kindergarteners’ letter-name knowledge, suggesting that early intervention for DHH children may include sufficient focus on naming letters and so targeted intervention is unnecessary.

#### Vocabulary.

Finally, *Foundations* appeared to be effective in improving children’s expressive vocabulary. Intervention children showed accelerated learning in both measures of expressive vocabulary compared to hearing norms, as well as compared to the comparison children. Interestingly, both intervention and comparison children showed similarly accelerated gains in receptive vocabulary. This latter result is consistent with two longitudinal studies that found DHH preschoolers increased their standard scores on the PPVT after enrollment in high-quality preschool programs (Hayes et al., 2009; Nittrouer, 2010). It may be that the typical, language-focused, early intervention for DHH children is sufficient to improve receptive vocabulary, whereas the language elicitation techniques included in *Foundations* provided additional opportunities to improve expressive vocabulary.

### Limitations

DHH children represent a low-incidence population; thus, conducting a strong group research design is challenging. There are several aspects of our research design that limit interpretations. First, we used a quasi-experimental design with no random assignment to intervention and comparison groups. This means that causal claims are limited. Second, the sample size was relatively small compared to typical intervention research. Given the diversity of DHH children, generalization from small sample sizes must be made with caution. Finally, the sample was collected over 4 years as part of an iterative design study. Although the framework and instructional strategies of *Foundations* remained the same for all 4 years, we made improvements based on teacher feedback and child performance. These improvements were primarily in the specific activities implemented rather than the type of activities (e.g., better letter–sound stories, better language activities). Even so, children did not receive exactly the same intervention for all 4 years. We combined the data across years to have a sample size large enough to conduct inferential statistics. Indeed, this is one of the only group design studies that can estimate effect sizes of an intervention on early reading skills of children who are DHH. However, the quasi-experimental design limited our ability to specify the exact intervention the children received and how it differed from that received by comparison children. Finally, the intervention was delivered by highly trained research teachers with almost perfect fidelity. At present, we are implementing *Foundations* with classroom teachers receiving coaching support to further extend our understanding of feasibility and generalizability of the results.

Importantly, only DHH children with functional hearing were included in this study. This was done for theoretical reasons: we hypothesize that learning to read is different for DHH children with and without functional hearing and decided to focus on DHH children with functional hearing because they form the majority of DHH children in school today (Easterbrooks et al., 2008). The extent to which these results will generalize to children without functional hearing is unknown and is currently under investigation. Single-case design studies suggest that instructional strategies designed to teach letter–sound correspondences are effective for DHH children without functional hearing, but the presently available strategies to teach phonological awareness may not be (Beal-Alvarez et al., 2012; Tucci & Easterbrooks, 2014). It is important to continue to investigate what instructional strategies can be used to best teach all DHH children to read.

## Implications and Conclusions

The NELP concluded that effective preschool interventions have moderate to large effect sizes on hearing children’s acquisition of phonological awareness, alphabetic knowledge, and vocabulary, and these interventions can prevent future reading problems for many children (Shanahan & Lonigan, 2010). The present study is the first to show that DHH children with functional hearing can acquire these foundational skills when taught with an intervention that is based on instructional practices found effective with hearing children but adapted to the unique needs of children who are DHH, specifically their decreased auditory access, weaker language skills, and wider individual differences. To address children’s decreased auditory access, *Foundations* provides experiences designed to build visual, kinesthetic, and semantic representations to supplement and support spoken phonological representations. To address children’s weaker language skills and wide individual differences, *Foundations* explicitly teaches a range of vocabulary within meaningful contexts. These explicitly taught words are also incorporated in phonological awareness activities, thus making these activities appropriate for children with smaller lexicons. The present study adds to a growing body of literature that suggests that preschool children who differ from typical hearing children can successfully learn to read with instruction specifically adapted to their needs.

## Supplementary Data

Supplementary material is available at http://jdsde.oxfordjournals.org/.

## Funding

Institute of Education Sciences; U.S. Department of Education (R324E06035, R324A110101 to the Georgia State University Research Foundation, Inc.). The opinions expressed are those of the authors and do not represent views of the Institute of Education Sciences or the U.S. Department of Education.

## Conflicts of Interest

No conflicts of interest were reported.

## Supplementary Material

Supplementary Data
