# Supplementary material for: Foundations for Literacy: An Early Literacy Intervention for Deaf and Hard-of-Hearing Children
Source: J Deaf Stud Deaf Educ. 2014 Aug 14;19(4):438–55. doi: 10.1093/deafed/enu022 (PMC4146385; doi:10.1093/deafed/enu022)
Supplement: Supplementary Data [file supp_19_4_438__index.html]

 Foundations for Literacy: An Early Literacy Intervention for Deaf and Hard-of-Hearing Children — Foundations for Literacy: An Early Literacy Intervention for Deaf and Hard-of-Hearing Children — Supplementary Data 

# *Foundations for Literacy*: An Early Literacy Intervention for Deaf and Hard-of-Hearing Children

## Supplementary Data

Data files

**Files in this Data Supplement:**

- Supplementary Data - Supplementary Data
