# Supplementary material for: Foundations for Literacy: An Early Literacy Intervention for Deaf and Hard-of-Hearing Children
Source: J Deaf Stud Deaf Educ. 2014 Aug 14;19(4):438–55. doi: 10.1093/deafed/enu022 (PMC4146385; doi:10.1093/deafed/enu022)

**Appendix A**

Miss Giggle Letter-Sound Story


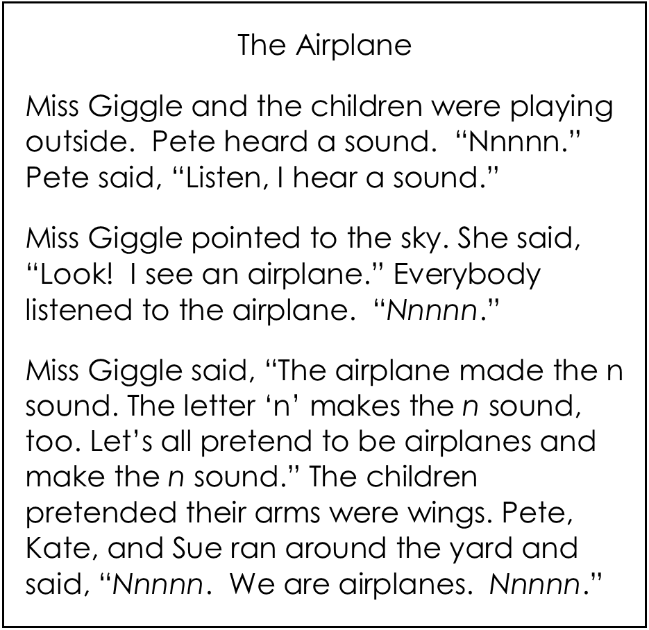


Story Sequence Cards


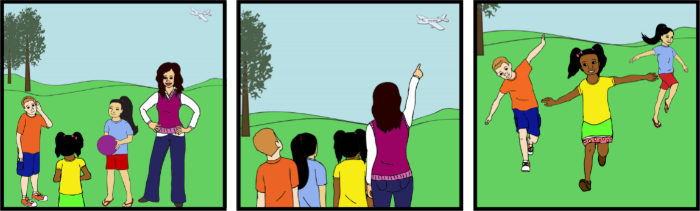


Large Sound Card


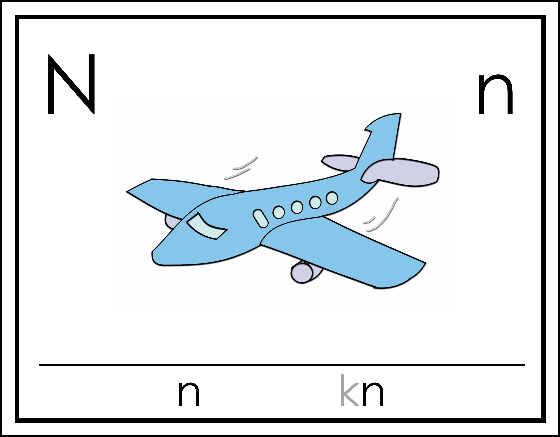


Small Sound Cards: b, o, n


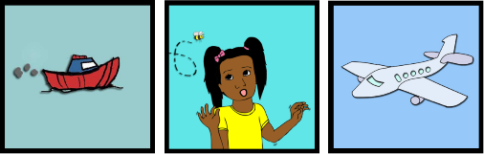


Vocabulary Picture Cards


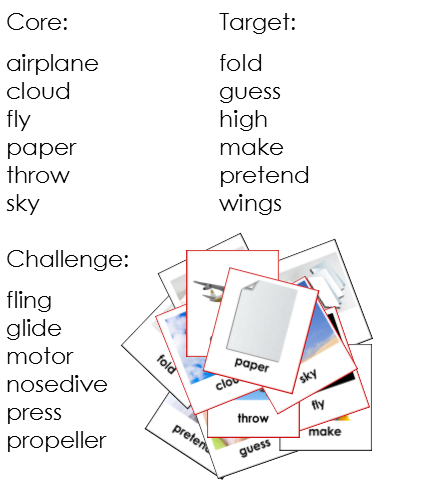


Vocabulary Words: Extension


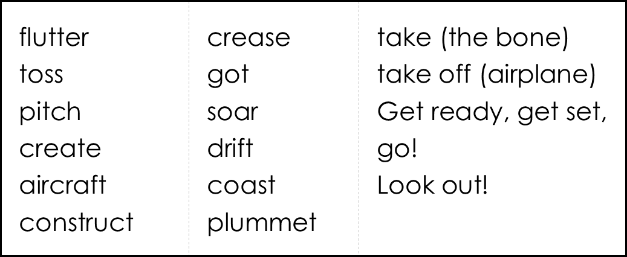


Letter-Sound Fluency Chart


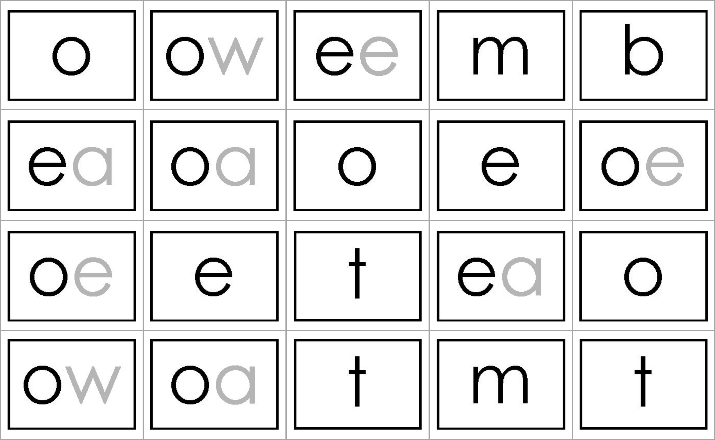


Decodable Word Language Activity


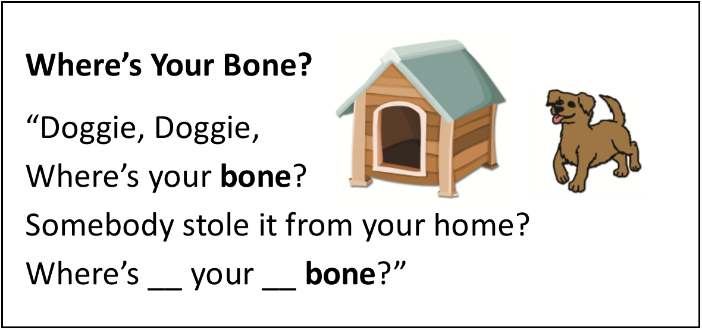


Decodable Word Blending


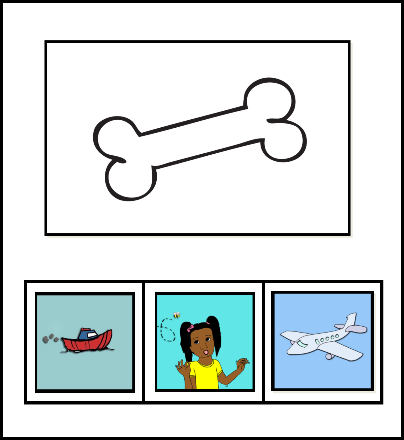


Practice Game: Decodable Word Bingo


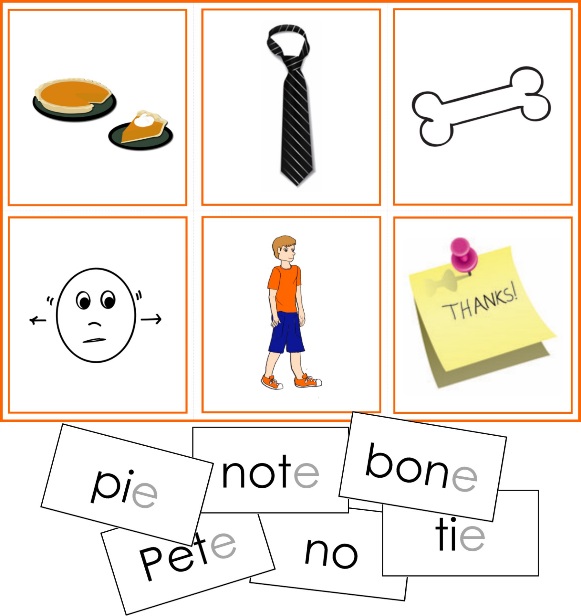


Note. Pictures are not to scale.

Phonological Awareness: Initial Sound


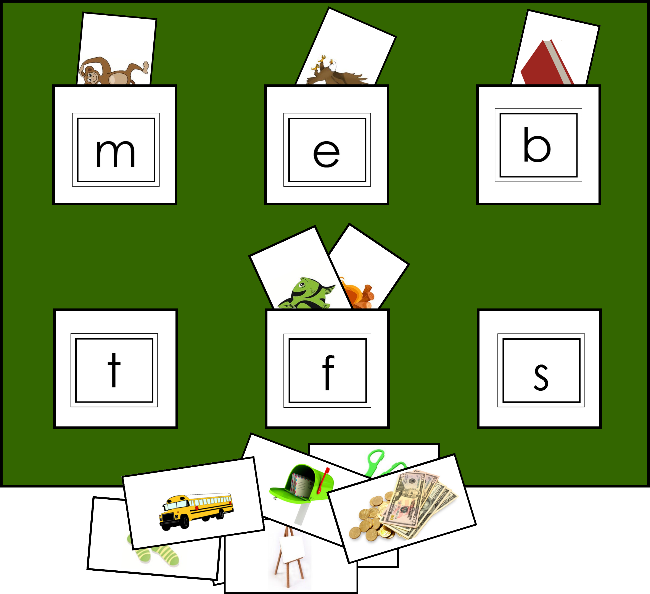

Supplement: Supplementary Data [file supp_enu022_Foundations_Appendix_A.doc]
